# China’s Land-Use Changes during the Past 300 Years: A Historical Perspective

**DOI:** 10.3390/ijerph13090847

**Published:** 2016-08-25

**Authors:** Lijuan Miao, Feng Zhu, Zhanli Sun, John C. Moore, Xuefeng Cui

**Affiliations:** 1 College of Remote Sensing and Geography, Nanjing University of Information Science and Technology, Nanjing 210044, China; 2 State Key Laboratory of Earth Surface Processes and Resource Ecology, College of Global Change and Earth System Science, Beijing Normal University, Beijing 100875, China; zhufeng314@mail.bnu.edu.cn (F.Z.); john.moore.bnu@gmail.com (J.C.M.); 3 Leibniz Institute of Agricultural Development in Transition Economies (IAMO), Halle (Saale) 06120, Germany; Sun@iamo.de; 4 College of System Science, Beijing Normal University, Beijing 100875, China; 5 School of Mathematics and Statistics, University of Dublin, Belfield, Dublin 4, Ireland

**Keywords:** land use transition, human-environment interaction, driving factor, China

## Abstract

Understanding the processes of historical land-use change is crucial to the research of global environmental sustainability. Here we examine and attempt to disentangle the evolutionary interactions between land-use change and its underlying causes through a historical lens. We compiled and synthesized historical land-use change and various biophysical, political, socioeconomic, and technical datasets, from the Qing dynasty to modern China. The analysis reveals a clear transition period between the 1950s and the 1980s. Before the 1950s, cropland expanded while forested land diminished, which was also accompanied by increasing population; after the 1980s land-use change exhibited new characteristics: changes in cropland, and decoupling of forest from population as a result of agricultural intensification and globalization. Chinese political policies also played an important and complex role, especially during the 1950s–1980s transition periods. Overall, climate change plays an indirect but fundamental role in the dynamics of land use via a series of various cascading effects such as shrinking agricultural production proceeding to population collapse and outbreaks of war. The expected continuation of agricultural intensification this century should be able to support increasing domestic demand for richer diets, but may not be compatible with long-term environmental sustainability.

## 1. Introduction

The coupled human-environment system is influenced by both biophysical and socioeconomic factors, making it notoriously complex and difficult to predict. The problem is even greater when regional land cover histories are poorly understood [1]. Global industrial development has significantly accelerated anthropogenic impacts on the land surface over the past 300 years [2], which has stimulated increased attention worldwide since 1990 [3]. Research on the long-term (decadal-centennial) history of land-use change (LUC) can provide distinctive insights of the complex interactions between society and environmental processes in land systems [4].

Reconstructed historical land use has served as the pivotal data for assessing general historical processes and trends of human activities at the global/regional scale and evaluating the long-term effects on biodiversity, biogeochemistry, geomorphic processes, and the climate system [3]. Usually, reconstructions and appraisals based on historical land-use are at the global scale where coarse approximations may be valid, with few regional studies, due to the lack of sufficiently accurate data on population and the spatial complexity and variability of human-environment interactions [5]. Globally, LUC reconstruction relies on inverse modeling, which incorporates dynamic changes in human-environment relationships over hundreds or thousands of years [6,7,8]. Regional historical reconstruction relies on archives, inverse modeling, or a combination of data and models [5,9,10]. A better understanding at the regional scales is relevant to our understanding of land-use change under a shared global environmental background but between different nation states.

China, as the third largest and the most populated country, has a long history of land cultivation and has maintained good historical records, particularly since 1700, despite various periods of destruction, including the Cultural Revolution [11,12]. Naturally in a country as large as China, and over a period as long as 300 years, there have been significant regional differences in LUC [5]. Previous studies have shown that there has been an increasing trend in cropland areas in China between 1700 and 1950, although they indicate different magnitudes and rates [5,13]. However, the considerable degree of political and social uniformity facilitated by generally easy topography, centralized government, and a common lingua franca made these regional differences far smaller than they were in other, similarly sized regions, such as Western Europe. Economically and socially, China was closed or semi-closed over our entire study period until the end of 1970s [14]. All these factors make China an exemplar to examine and disentangle the evolutionary interactions between land-use and its associated driving factors through a historical lens. In this study, we synthesize available data on land-use, and attempt, on a macroscopic level in Section 2, to show how the LUC in China has evolved over the past 300 years and, more importantly, how it has been shaped by the underlying drivers, such as population, climate change, agricultural intensification, national policy and globalization.

## 2. Materials and Methods

The historical datasets of Chinese land-use and its associated factors were obtained from a selection of multidisciplinary sources, and included demographic, agricultural, forestry, sociologic and political records. We screened all the available datasets known to us that cover the past 300 years and, where possible, preferred original records and data rather than reconstructed datasets in this study. The data selection process is described in the supplementary materials.

### 2.1. Population

**Figure 1:**
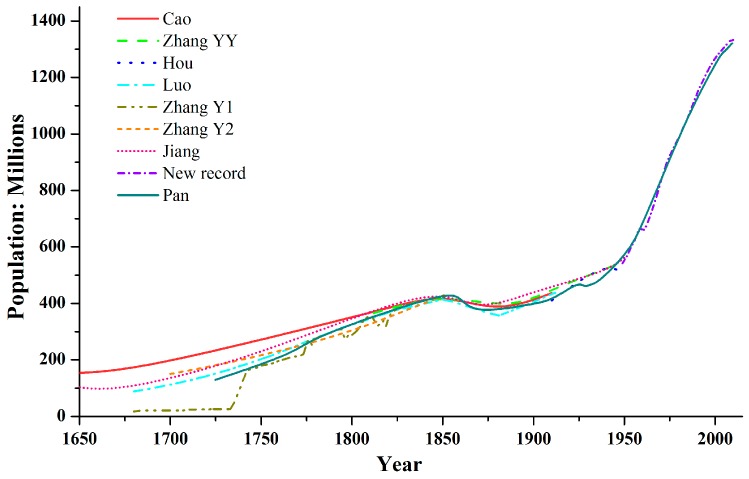
Historical changes of the population. Data resources: Cao [16], Zhang Y.Y. [17], Hou [18], Luo [19], Zhang Y1 & Y2 [20], Jiang [21]; New record: the Statistics of the Official Population in China since 1949, Pan [15].

A substantial number of datasets have previously been used to describe the Chinese population over the past 300 years (Figure 1). It is clear that these datasets differ in absolute numbers, but show similar temporal variations. Perhaps the most reliable estimate was recently reported by Pan et al. (2013) who built the population database from revised firsthand records over 286 time periods [15]. After the founding of the People’s Republic of China in 1949, the data is based on the six national population censuses in the years 1953, 1964, 1982, 1990, 2000, and 2010. These census data are generally regarded as more accurate than the historical reconstruction used before 1949.

### 2.2. Cropland

**Figure 2:**
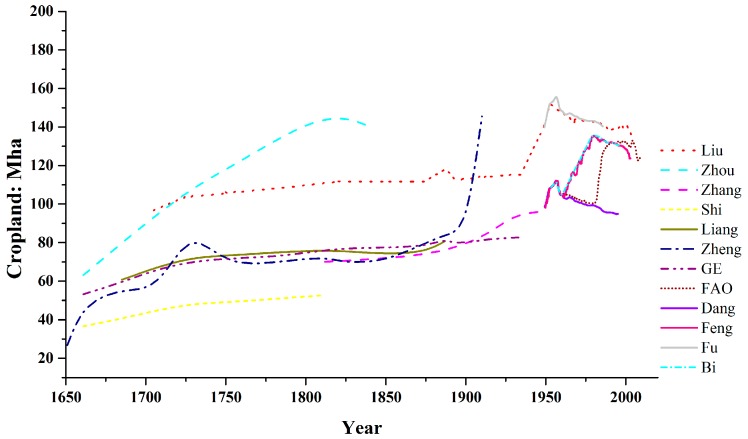
Historical changes of the cropland area. Data resources: Liu [23], Zhou [24], Zhang [17], Shi [25], Liang [26], Zheng [27], Ge [11,13], FAO (Food and Agriculture Organization of the United Nations) (1961–2009), Dang [28], Feng [22], Fu [29], Bi [30].

Various published estimates on cropland area are plotted in Figure 2. The cropland statistics are less consistent between datasets than population. Here we preferred a study based on 3 northeastern and 18 other provinces in China [11,13] for cropland variation during the Qing dynasty and the Republican period (that is, prior to 1949). This data is highly continuous and close to the median of the different datasets (Figure 2). After 1949 we took governmental statistics on cropland area [22].

### 2.3. Forest Coverage

Chinese forestry datasets are fewer than for population or cropland datasets. For data prior to 1949, we deferred to [31], whose data is derived from historical documents, surveys, and statistics and also from earlier studies. For data after 1949 we used the forest resource inventory of China, which reported forest coverage in several periods (1973–1976, 1977–1981, 1984–1988, 1989–1993, 1994–1998, 1999–2003, and 2004–2008) and includes detailed definitions of survey methods and technologies [5,32,33,34].

### 2.4. Climate Index

**Figure 3:**
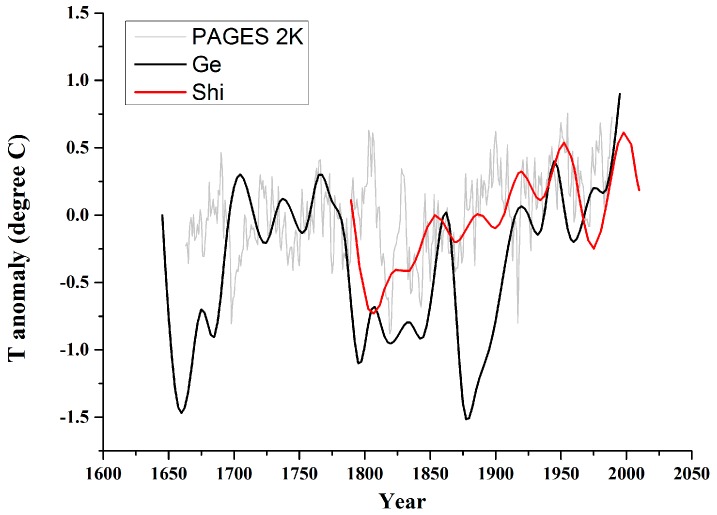
Temperature anomaly reconstructions: PAGES 2K representing Asian temperature [35]; Ge representing central and eastern China winter half-year temperatures based on historical archives [36]; Shi representing southeastern Tibetan Plateau summer minimum temperature [37].

We used three datasets to describe the historical climate change trends over the past 300 years, (1) PAGES 2K: tree ring reconstructed temperature anomalies for Asia [35]; (2) Ge: winter half-year (October to April) temperature anomalies reconstructed over the past 300 years in the main crop areas in eastern China based on 200 phenological and crop records, winter snow days and historical meteorological recordings [36]; and (3) Shi: East Tibetan Plateau summer minimum temperature anomalies inferred from Alpine tree line dendrochronology [37]. High resolution regional climate models show that most of China responds similarly to climate change [38], though variability in precipitation is larger than for temperature. Figure 3 illustrates that the Ge and Shi records show generally similar features, though the magnitude of the variations varies, as may be expected given that they are records representing mainly winter conditions in eastern China and summer conditions in western China, respectively. All three records show similar warming trends since 1800 of about 0.4 ± 0.1 °C/century. Using a single climate index for a country as large as China is of course an approximation, and all three indexes also rely mainly on proxy data rather than being instrumental records. The temperature data from Ge et al. (2003) was chosen for further studies as it mainly based on historical archives, many of which are directly related to agriculture [36]. Since drought severity may be expected to play a role in limiting agriculture in China, we also used a drought index reconstructed from historical records [39]; this index suggests more severe drought events than the Monsoon Asia Drought Atlas (MADA) [40], which was based on tree rings.

### 2.5. Agricultural Production Index

We used an index that quantifies production on a scale ranging from 1–10 for the period before 1949 [41]. The production index was normalized to Qing dynasty harvests with 10 meaning a generous harvest and 7 a normal harvest. Between 1949 and 2008 we took yield records from the Ministry of Agriculture of the People’s Republic of China.

### 2.6. War Frequency

War frequency has been used as a simple proxy for the intensity of damage caused by conflicts both globally and locally [42,43]. The data we used in this study was based on literature collected in the Chinese imperial war chronology, which records the timing, locations, causes and impacts of wars and was prepared by the Editorial Committee of Chinese Military History in 1985. Zhang et al. (2007) pointed out that although different sources might not agree on number of wars due to the difference in the definition of a war, e.g., numbers of troops or casualties, the various source records showed a common pattern in the number variability over time [42]. Zhang et al. (2007) also showed that there was a clear correlation between war frequency and climatic stress (mainly due to cold arid periods) [42]. For example, between A.D. 1620 and 1650, the Chinese population fell by 43% (70 million people) because of wars, starvation and epidemics [21]. Therefore, we concluded that war frequency demonstrating the instability of society has a great impact on population and agricultural practice.

## 3. Results

### 3.1. Historical LUC Patterns in China

**Figure 4:**
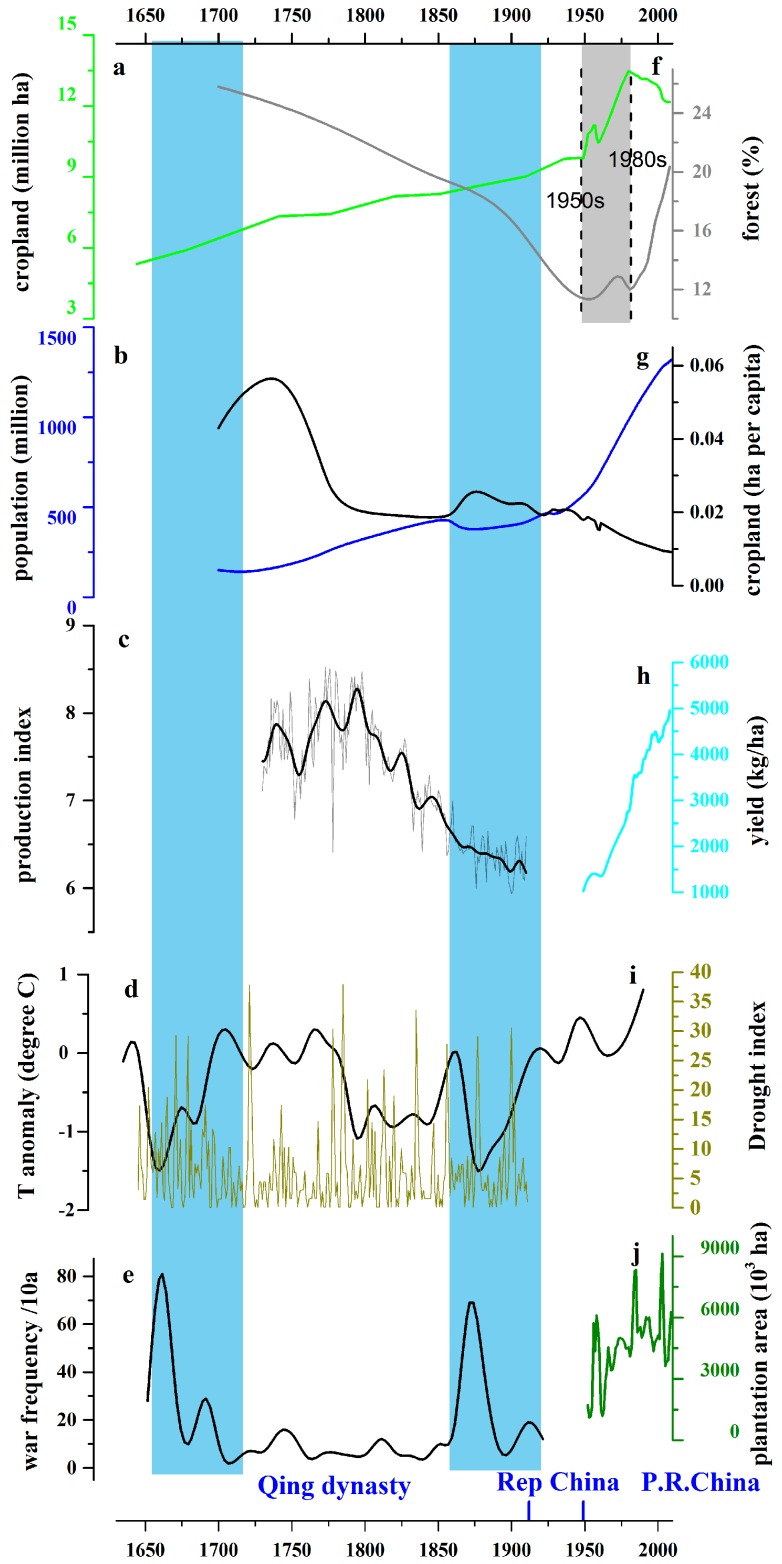
Chinese land-use changes and associating factors during the past 300 years. (**a**): cropland area; (**b**): population; (**c**): production index (**d**): annual average temperature (**e**): decadal war frequency; (**f**): forest coverage rate; (**g**): cropland per capita; (**h**): yield since 1949; (**i**) Drought Index; (**j**) tree plantation area since 1949. The blue shadow sections indicate relatively cold periods, the grey shadow highlights the transition period. This figure was reused from Cui et al., 2015 [49].

Figure 4a shows LUC in China can be divided into three distinct episodes: 1650–1949, 1949–1980s, and the 1980s until now. From 1650 to 1949 the area of cropland generally increased, while the forest cover decreased. Over the long history, approximately half of cropland expansion came from deforestation in China [23]. The remaining cropland came from recultivating the land abandoned during wars and conversion of wetlands and barren land [44]. Cropland spatial distribution shows large regional differences between provinces: the major centers of agricultural production provinces are located in eastern China, Shandong, Henan, Anhui, Hunan, Hebei, and Jiangsu. The greatest increases in cropland area occurred in northeastern China, specifically, in the three provinces of Heilongjiang, Jilin, and Liaoning, but also in more southerly provinces Henan, Jiangsu, and Anhui [5].

However, since the 1980s, cropland area has generally decreased, while forest cover increased, which is mainly the result of plantation projects. The period between 1949 and the 1980s was an unstable period, exhibiting large variations in land-use and also considerable uncertainties in the data. This period is also characterized by inconsistent administration in China, and is dominated by several different and opposing land-use policies. After the implementation of the land system reformation in 1952 and the establishment of the socialist system in 1956, the government abolished the traditional and exploitative feudal land system; land-use rights were given to farmers, although the state maintained ownership of the land [45]. The Great Leap Forward (1958–1960) was an attempt to leap ahead in production by reorganizing the peasantry into large-scale communes and mobilizing society to produce a technological revolution in agriculture, resulting in millions of workers moving into cities to work in factories [46] and widespread famine. Subsequently the Cultural Revolution (1966–1976) saw millions of well-educated people sent to the countryside as farm workers; this in turn improved rural education and health care, preparing the ground for the later reforms [47]. The Great Reform and Opening-up Policy in 1978 included modification of land management, economic structural adjustment, and political restructuring to reverse the negative impact of the former policies and much more attention was paid to protection of cropland areas [48]. Each of these policies successively dominated land-use, and each was implemented nationwide.

### 3.2. Drivers of LUC

#### 3.2.1. Population

As shown from Figure 4b, population in China experienced a long slow growing period before the recent eruptible jump. To be more specific, it first maintained a fast and stable growth during 1724–1852. During 1852–1870, there were an obvious decrease which might relate to pestilence and famine, the Taiping Rebellion (1851–1864), and the Second Opium War (1856–1860) [49]. After 1870, the slow steady growth recaptured until 1960. In recent years, the growth rate tends to slow down due to the implementation of the “one child policy” [15].

Among all the driving factors discussed here and shown in Figure 4, population shows the best fit in phase of cropland changes and opposite phase of forest changes in general. It is easy to understand that the impact of population on cropland as population growth contributes to both increased local and import demand for food and other natural products [1,50] as explained by the theory from Boserup [51]. The long term agricultural development history may be used to explain the relationship between population and land-use: a causal connection dominates the quantitative dynamics of “population-cropland-forest”—the area of cropland increases and the area of forest decreases with the growth of the population [49]. If productivity improvement is not readily available while uncultivated land is, then the most immediate way to increase food production is via expansion of cropland, which generally occurs at the cost of natural forests and unutilized land. Such dynamics have been observed in many places around the world [49]. This can also be found in other regions, for example, a significant positive correlation existed between cropland area and population during 1910–1950 in South Africa [52], and it has been used in the reconstruction of global historical land-use by many scientists [6,7,53].

#### 3.2.2. Agricultural Technology and Intensification

Disputes remain regarding the quantified grain output of cropland in the early and mid-Qing dynasty [54], but it is generally agreed that agriculture had developed to a considerable level in the Qing dynasty and was superior to that of former dynasties in China [12,13]. Thus, agriculture improvements provided sufficient food and materials to sustain the unprecedented and enormous increase in population during the “Kang-Qian Golden Age (1681–1796)”, which lasted for more than 100 years. During this period, the increase of yields mainly relied on the improvement of the cropping regime, which was manifested by the introduction and the spread of new crops from the Americas and the increasing prevalence of multiple cropping (the planting of two or more crops in the same field in one year) [55]. Because of their adaptation to harsh environments and their relatively high return on investment, New World crops (for example: *Zea mays* and *Solanum tuberosum*) were introduced in China during the Ming and Qing dynasty and were completely embraced by the Chinese farmers. The new crops gradually spread out and even grew in the barren lands of arid hills and poor-soil mountains, which otherwise could not be utilized for indigenous crops. The other approach that greatly contributed to the increase of the yield is the increase in multiple cropping. During the Qing dynasty, the overall multiple cropping index (the sum of areas planted to different crops harvested during the year, divided by the total cultivated area) of China surpassed one [56]. In the southern part of China, where rice has been traditionally single-cropped during the Ming dynasty, double-cropping practices were promoted and became widespread [57].

Cropland per capita (Figure 4g) is a good indicator to show agricultural intensity. The decrease of cropland per capita indicates that the rate of cropland expansion was not as rapid as the rate of population growth. This normally means that either cropland expansion became increasing difficult or food productivity increased largely. Research showed that limitations to land expansion occurred as early as the time of Emperor Jiaqing (1796–1820), when little fertile land was left for reclamation in the inland regions of China leading to exploitation of more marginal and remote lands [12,58,59]. Before 1968, China could feed the increasing population even when the per capita ratio of cropland was not very high [60]. However, in recent times, the declining trend of the cropland-population ratio raised food security concerns as both population pressure became increasingly severe and changing diet increased demand for a larger variety of agricultural products per capita [61]. The declining ratio of cropland-to-population indicated that the reclamation of new land had become increasing difficult and that cropland expansion could not address both population growth and the land limitations. In this context, satisfying the need of the growing population of China by increasing the food production must rely more on increases in productivity than it had during the past three centuries. During most of the historical period most of the people remained at the subsistence level. The increase of yield resulting from the agricultural intensification, especially after 1949, supported the enormous population growth in China.

The increase of yield per unit land in Figure 4h results from the agricultural intensification, which is generally perceived as the outcome of technological progress. Systemic and intensive agriculture burgeoned as early as the Eastern Zhou dynasty (771BC-221BC) in China [56]. Typically, for most of Chinese history, the development of agricultural technology progressed slowly. During the last 100 years, agricultural tools that were designed 1000 years ago were still being used, and cultivation techniques depended on experiences passed on from generation-to-generation. While China achieved a yield increase through non-technological means in the traditional period before 1949, it continued to achieve yield increases with the help of modern technologies—such as fertilizers, pesticides, and mechanization—and via institutional reform, that is, the household responsibility system introduced in the early 1980s, especially after the Great Reform and the Opening-up Policy started by Deng Xiaoping. In 2012, China’s grain production increased for the ninth consecutive year, for the first time in history, and technology accounted for more than 85% of this increased production [62]. Besides agricultural incentives from the government, this increased production has to be attributed to the change of land-use practice, which directly led to the modern land-use intensification.

#### 3.2.3. Climate Change and Interactions among Driving Factors

Population and land-use is an integral system throughout history; other factors that make the analysis of the driving forces of land-use more complicated include climate change and wars. There are two temperature valleys between 1650s–1670s and 1870s–1880s in China, according to the reconstruction by Ge in Figure 4d. The Qing Dynasty (1644–1911) was in general relatively cold, when China experienced a “Little Ice Age” [43]. During the Qing dynasty, the decreasing food production, high population growth rate, and increasing numbers of wars corresponded with the colder periods (see Figure 4c,e,g). During the coldest periods, around the 1850s and the 1870s, China was (perhaps not coincidentally) undergoing periods of social unrest, experiencing a dynastic transition and civil rebellions, respectively, as shown in Figure 4d,e. During the relatively warmer period occurring about 1700–1800, China experienced the “Kang-Qian Golden Age” (also called “High Qing”) with low war frequency and stable population increasing, which has been highly praised by historians for its economic success [63].

The direct negative effect of cold weather may cause shrinkage of agricultural production, consistent with a shortened growing season as shown in Figure 4c,d from about 1850s–1870s. In the agrarian society of ancient China, a decrease of agricultural production resulted immediately in food shortages, fundamentally due to a lack of organized social buffering mechanisms. When the food supply was insufficient, social conflict and war tended to break out as an adaptive, but inefficient and unsustainable, method of resource redistribution [44,64]. Historical accounts during these times are filled with scenes of abandoned farms along with deserted villages and towns after wars [65,66]. Since the 1970s, Shi et al. (2014) indicated that climate warming has provided the thermal conditions that have aided rapid cropland reclamation, whereas previously climate was a limiting factor in northern China [67]. The history of rice cultivation in the middle and lower reaches of the Yangtze River suggests that the adoption of a double-cropping system in this region was largely synchronous with the warm phases [55], supporting the notion that the environment must first preconditioned to support agricultural intensification processes, rather than it being purely due to human effort. Climatic extremes such as severe droughts also show strong (lagged) anticorrelation with production, with the period of few droughts in the mid-18th century (Figure 4i) leading up to the peak of production index (Figure 4c), and the increased drought index afterwards occurring during declining production. There is no clear centennial trend in the frequency or severity of droughts, but we can conclude that severe drought events impact heavily on agricultural production.

It is difficult to determine how and to what extent climate change rather than the human population pressure has affected land-use. It is clear that there is feedback between climate, population and agricultural production, and root cause and proximate causality relations require highly resolved data to determine. Zhang (2011) concluded that climate-driven economic crises directly caused significant human crises in preindustrial Europe and the northern hemisphere, based on the statistical analysis of fine-grained historical data [64]. Thus, when analyzing the historical land-use dynamics, the population and climate should be viewed in comparable perspective.

#### 3.2.4. National Policy

Historically, China has always had an effective central government in most of the past 2000 years [68]. As a result, national policies on agriculture, forest and land resources played a vital role in shaping the land system in China by, for example, enforcing land institutions, implementing taxes or subsidies on agricultural production, regulating land markets, and directly redistributing land for different uses [69,70,71,72]. The family-based small-holder system has been the dominating farm structure in Chinese agriculture production, except when interrupted between the 1950s and 1979 during land nationalization and collectivization under the communist movement [73]. Farm structure and land institutions have profound implications for agricultural production and land-use. Before 1949, most arable land was owned by a small percentage of rich and/or powerful people, although the majority of the land was rented to small holders or cultivated by hired laborers. Agricultural land expansion, usually into forests, was the major strategy for increasing agricultural production because small farmers seldom had sufficient capital for new technology or machinery. Large land owners maximized profit by either expanding cropland if sufficient laborers were available, or occasionally by intensifying the production by, for example, adopting new agricultural technologies. During the 1949–1980 period communist policies, including the grain-first campaign, the Great Leap Forward, and the Cultural Revolution along with collectivization led to dramatic changes in agriculture production and natural resources management; as a result, agricultural land greatly increased at the cost of, for example, wetland and forest land [74,75,76]. After 1979, the Household Responsibility System was implemented and restored the small-holder structure which contributed greatly to the sharp increase in agricultural production [77]. Additionally, the agricultural intensification fueled by a national emphasis on investment in agricultural technologies also simulated significant agricultural production growth [78]. This in turn paved the way for the shifting of national land policy towards ecological restoration [79] and facilitated the decrease of cropland as observed in Figure 4. Most recently, the land transfer and circulation policy permitted land cultivation rights to be transferred, and is a strategy designed to increase land efficiency through land consolidation, but its effects remain to be observed [80].

The areal decline of cropland has been driven by two factors: (1) massive and rapid urbanization fueled by economic development since the 1980s; and (2) reforestation and afforestation supported by large-scale environmental protection programs. The urbanization level in China increased from 17.9% in 1978 to 40.5% in 2003, demonstrating a growth twice as fast as the world average over the same period [81]. The urbanization process transformed large areas of agricultural land around cities and towns to urban land-use, especially in eastern China. According to Liu (2005), urbanization accounted for 46.1% of total national cropland loss, that is, 1.12 Mha during the 1990s [52]. In more developed regions, such as north, southeast and central China, urbanization was the dominating factor for the decrease of cropland loss. After the year 2000, this pattern continues but the pace of urbanization is even faster [82]. The areal reclamation of forest in China, termed the forest transition [83], was not, in the main, due to the natural forest reforestation and forest regeneration on abandoned cropland, but rather it was largely due to massive tree plantation efforts supported by national forestry programs and environmental policies [84] that were initiated since the 1980s, after Chinese economic growth began.

The Chinese government launched a series of ecological projects, such as the Natural Forest Protection Program (NFPP) and the Sloping Land Conversion Program (SLCP), to fight against sandstorms, flooding, or droughts; such natural disasters have caused enormous economic damages in China in recent decades [85]. These ecological projects are among the largest programs in the world as measured by area, investment, and potential ecological impacts [84]. The total tree plantation area increased significantly after the 1980s, as shown in Figure 4f,j, becoming as high as 62 Mha, and accounting for 34% of Chinese total forest area. In view of the forest transition in the 1980s, and the foreseen increasing forest cover in future, China seems to have “bent the curve” of the forest use trend in a favorable direction [86], largely due to agricultural intensification and institutional intervention. However, some constraints and tradeoffs, for example, logging old-growth forests, changes in fires, and insect outbreaks under changing climate, must be carefully considered and addressed before we can anticipate a bright future [87].

## 4. Discussion

### 4.1. Chinese Land-Use Transition

The history of Chinese land-use over the past 300 years demonstrates how the population strived to increase exploitation of land resources to sustain its own population growth [49]. If we ignore some short period fluctuations, Figure 4a,b,f show that the increase of cropland area kept pace with the population growth, and was related to continuous forest decline before 1949. However, the prominent feature in Figure 4a,b,f is that after the 1980s, the population growth continued to increase while cropland area began to decline and forest coverage started to increase. Therefore, the historical “population-cropland-forest” relationship seen in China over the previous 300 years underwent a transition during between the 1950s and the 1980s [49]. During the transition period, the land expropriation conflicts between the nation and households as a result of the immature land-use law, the defects of the land-use system, and the characteristics of natural resources seem to be a very serious problem in China, especially in the village region [88,89]. Also, the urbanization speeded up from 1949 to 1970, and the conflict between the cropland and urban area seems to be irreconcilable [90].

Cropland could only provide food at the subsistence level for almost all the population before 1949. With limited access to technology and increasing population, farmers sought to expand cropland as the means to increase food production. In the New Period after the 1980s, overall Chinese consumption increased faster than before due to both the rising population (which was slowed but not reversed by strict family-planning policies) and, more importantly, by the changing diet per capita, which increased significantly due to rising income of the population as a whole. With more financial resources available, farmers were able to adopt modern technologies (including irrigation infrastructure, mechanization, fertilizer, and pesticides). This became a better option for increasing productivity than cropland expansion as national environmental protection policy became stricter. The result is that China is self-sufficient in terms of food production, despite consumption increases.

### 4.2. Dynamics of Land-Use Transition in China

There are many views of the links between population fluctuations, food supply, and climate. Clearly there must be political, physical, and technological factors involved in the relationship between food supply and population. This relationship may be one of calamitous population swings as populations rise and are checked by war and famine, or potentially stable under steady state environmental conditions, or stably rising with technological growth. This pattern prevailed in China before 1950s and seems consistent with Malthusian theory, which was established from the similar pattern observed between 1600 and 1900 in Europe. In China, the population collapsed in the late 19th century, concomitant with diminishing agricultural production and cropland per capita, reduced population, and low temperatures. In contrast, after the 1980s, as depicted in Figure 4h, the Boserupian process [51,91] appears to dominate as China managed to sustain the booming population via increasing agricultural intensification, for example, by adopting the new agricultural technologies developed since the Green Revolution of the late 1960s. This practice was delayed in China, until reform of institutions that had constrained agricultural production through the transition period of the 1950s–1980s. Hence it is clear that both Malthusian and Boserupian views are incomplete since they do not allow for the importance of the political control of land-use. Over longer Chinese history, Zhang et al., (2007) note that the Boserupian view is only consistent with their observations of population change over periods as long as millennia [42]. That decadal and centennial scale climate change can create food supply pressures that result in war and population decline that appear Malthusian. In contrast, the profound political and social changes of China from the 1950s–1980s appear to denote a land-use regime shift, averting the disaster of falling deep into a Malthusian trap. However, the relatively short period since the 1980s hardly passes a long-term stability test, and relies on maintaining an increasing rate of agricultural productivity.

### 4.3. Future Perspectives

Will the benefits of agricultural intensification be sustainable and will the yield increase continue in the future? These questions are not only relevant for China but also for the sustainable development of the world. Intensification of agriculture by using high-yielding crop varieties, fertilizers, irrigation, and pesticides has contributed substantially to the tremendous increases in food production over the past 60 years [92]. Agricultural intensification has been advocated as one of best means to address the tradeoff between food production and environmental conservation. The increases in cereal yields have saved natural ecosystems from being converted to agriculture in general, and agricultural intensification has been seen as a cost-effective means to avoid emissions of greenhouse gases [93,94,95]. In contrast, intensification and yield increase may produce a “Jevons paradox”, whereby improved resource efficiency leads not to reduced resource consumption, but instead greater overall use [95,96]. The existence of the “Jevons paradox” in reality may tarnish our vision that intensification would spare more land for conservation by concentrating agricultural production on existing farmland without further expansion. In the tropics, agriculture has generally intensified and yield increased over the last few decades, but deforestation has continued, mainly due to conversion of forest to agriculture [95,97]. The agricultural intensification from 1990 to 2005 was not generally accompanied by decline or stasis in cropland area except in countries with grain imports and conservation set-aside programs, such as China [98]. Agricultural intensification has also been blamed for rising land rents, resulting in escalating future conservation costs, and for various environment problems related to the overuse of fertilizers and pesticides [92], such as alien plant invasions [99], increased risk of zoonotic disease emergence [100], and bird population declines [101].

Land-use systems in modern society are also affected by the globalization process. The historically strong relationship between domestic population growth and land-use change may become disconnected by globalization due to, for example, food imports [102]. Since the 1980s, the economy, including the agricultural system of China, has been increasingly integrated in the global system. Food imports have been steadily increasing [103], which relieves the pressure of domestic food production and cropland areal maintenance or expansion. The import of soybeans, for example, allows the cropland in northeastern China to be switched to high-yield corn and wheat that demands less water utilization, which has contributed to increased grain harvest [104,105].

As the most populous country, China’s food security has always been a global concern. After the establishment of the People’s Republic of China (PRC) in 1949, food security was a major preoccupation for policy makers [106]. In 2006 the Chinese government set a “red line” of 18 billion mu (1.2 billion ha) of cropland to ensure food security. In 2013, the most recent national land-use survey reported that some 1.33 billion ha of the country was planted with crops, though some have questioned the quality of the cultivated land [107]. Although over recent decades, yield has significantly increased in China, its rate of increase has clearly slowed. The yield may be approaching a ceiling, although China’s grain production increased for the ninth consecutive year in 2012, for the first time in history. The huge demand for food in China will continue to increase due to rapid economic development, accelerating urbanization, and changing diet. Also, it will impose more pressure on both the agricultural base and the environment, both at home and abroad [108]. Continuing population and consumption growth will increase the global demand for food for at least another 40 years [61], and perhaps through the whole century [109]. If the Chinese food security issue cannot be solved, the increasing popularity of meat- and dairy-based diets will put severe pressure on the global food market [110].

## 5. Conclusions

We have discussed the dynamics and various factors associated with China’s land-use from 1700 to 2010. Historically, we showed that as cropland area increased, in general, forest area decreased until 1949, when the situation reversed during a transition period from 1950 to 1980. This indicates a paradigm shift and the beginning of a new land use era in China. Population, climate change, new technology development, agricultural intensification, government policy and globalization were discussed as prominent proximate and underlying driving forces that have shaped the long-term land-use trajectory. Historically, climate change, wars, population, and land-use were strongly interrelated and may also shape future pathways. Since the unification of China, various national agricultural policies, and the increased availability of technology coupled to a generally ameliorating climate have dominated land-use change. We argued that population is the ultimate underlying driving force of LUC but its effect on land-use has been dwindling as the increased productivity from agricultural intensification made it possible to support more people with less land. Thus, agricultural intensification made it less urgent and less necessary to expand the amount of land for food, providing the necessary condition for the land-use transition. Government policy since the 1980s aimed to reverse deteriorating environmental quality and acted as the direct driving force for land-use after the transition period from 1950 to 1980. However, whether agricultural intensification can be sustainable in the long term is highly questionable. Mounting environmental issues, such as carbon emissions, water and soil pollution, and depleted biodiversity are linked to intensive food production. Thus in addition to the pressure of maintaining food security in the face of increasing consumption, the intensification of agricultural production also threatens the sustainable development of China.
